# Oncolytic virotherapy against lung cancer: key receptors and signaling pathways of viral entry

**DOI:** 10.3389/fimmu.2024.1473288

**Published:** 2024-10-04

**Authors:** Wenxun Dong, Ying Luo, Daqian He, Ming Zhang, Jingtong Zeng, Ying Chen

**Affiliations:** ^1^ Department of Thoracic Surgery I, Peking University Cancer Hospital Yunnan, The Third Affiliated Hospital of Kunming Medical University, Yunnan Cancer Hospital, Yunnan Cancer Center, Kunming, China; ^2^ Institute of Medical Biology, Chinese Academy of Medical Sciences, and Peking Union Medical College, Kunming, China

**Keywords:** lung cancer, oncolytic virus, virotherapy, viral entry receptors, signaling pathways

## Abstract

Lung cancer accounts for the highest cancer-related mortality worldwide. While immunotherapies targeting anti-tumor immune responses have demonstrated efficacy in clinical practice, the demand for novel treatment modalities remains urgent. Oncolytic viruses (OVs), which selectively kill tumor cells while stimulating an anti-tumor immune response, represent a potential breakthrough in lung cancer therapy. The induction of anti-tumor immunity by OVs is central to their overall therapeutic effectiveness. Many natural receptors on the surface of cancer cells are dysregulated, providing potential entry points for OVs. Furthermore, the inherent dysregulation of some key signaling pathways in lung cancer cells promotes proliferation, progression and metastasis, which may facilitate selective viral replication. In this review, we explore the application of OVs in lung cancer by analyzing several major OVs and their corresponding entry receptors. Then, we also examine the key signaling pathways and molecules with the potential to synergize with OVs in modulating the immune tumor microenvironment. Finally, we discuss the combination and administration strategies that warrant further clinical trials for validation. Despite certain limitations, the tolerability of OVs positions virotherapy as a promising avenue in the future of lung cancer treatment.

## 1 Introduction

### 1.1 Evolution of lung cancer treatment modalities

Lung cancer is among the most prevalent and deadly cancers globally, affecting countless individuals and families across various regions (1). In 2022, the International Agency for Research on Cancer (IARC) estimated 20 million new cancer cases worldwide, with lung cancer accounting for 12.4% of cases and 1.8 million deaths, the highest among all malignant tumors (2). Lung cancer is broadly classified into two types: small cell lung cancer (SCLC), representing 15% cases, and non-small cell lung cancer (NSCLC), accounting for 85% (1, 3).

**Figure 1:**
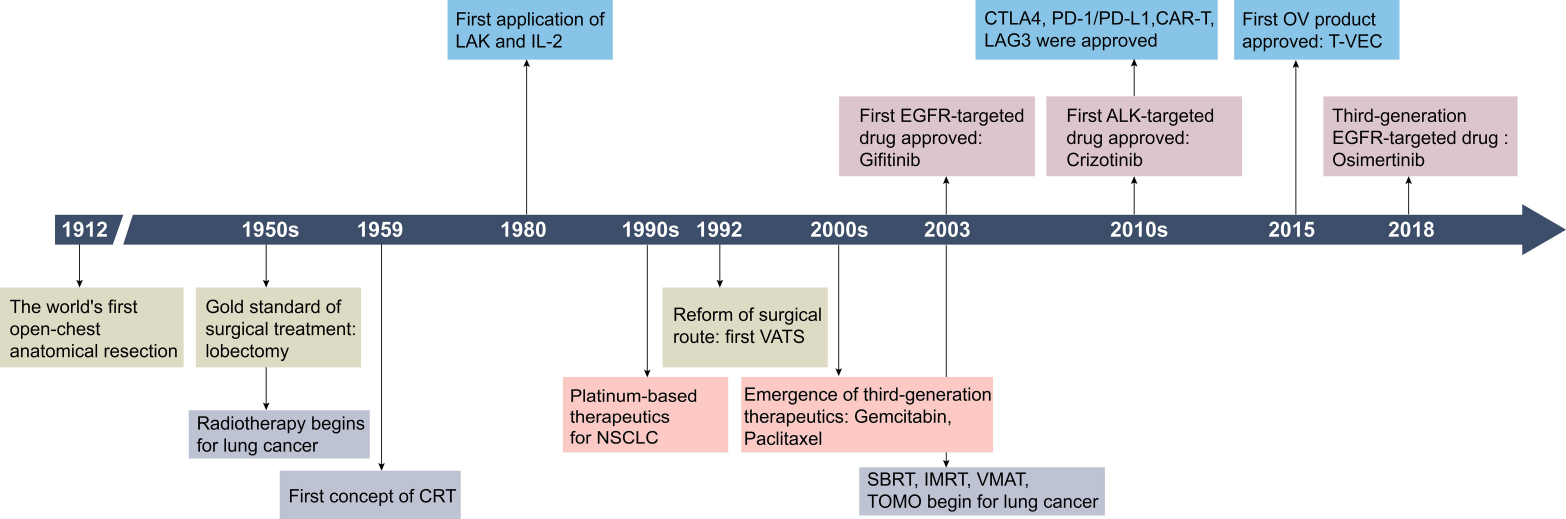
Historical timeline of key developments in lung cancer treatment strategies. CRT, 3D-comformal radiotherapy; LAK, lymphokine-activated killer cells; IL-2, interleukin-2; VATS, video-assisted thoracic surgery; SBRT, stereotactic body radiation therapy; IMRT, intensity modulated radiation therapy; VMAT, volumetric modulated arc therapy; TOMO, tomotherapy; CTLA-4, cytotoxic T lymphocyte antigen-4; CAR-T, chimeric antigen receptor T-cell immunotherapy; LAG3, lymphocyte activation gene-3. Created by Adobe Illustrator 2024.

Advances in lung cancer treatment have significantly expanded therapeutic options (**Figure 1**). For patients with stage I-II and select stage III NSCLC, the standard surgical procedure is lobectomy and mediastinal lymph node dissection, often supplemented with adjuvant radiation therapy or chemotherapy as needed (4, 5). In advanced NSCLC or SCLC, comprehensive treatment with targeted therapy and immunotherapy have become essential (4). Early intervention is associated with improved outcomes, while the five-year survival rate for patients diagnosed at advanced stages ranges between 4% and 30%fluctuating between 4% and 30% when they are diagnosed in the middle to advanced stages (6, 7).

### 1.2 Oncolytic virus therapies: new options in cancer treatment

Over the past century, significant advancements have been made in lung cancer therapies. However, the biological complexity and heterogeneity of lung cancer cells present challenges for conventional treatments. Since the advent of precision medicine in the 21st century, molecular profiling of tumors and immune cells has increasingly been used to guide therapeutic decisions (8). As a form of tumor immunotherapy, viral therapy shows great potential in lung cancer treatment by selectively killing cancer cells and activating antitumor immune responses (9). The use of pathogens in cancer treatment dates back to the late 1800s, when Dr. Coley used bacterial toxins to treat solid tumors (10). Additionally, there are reports of leukemia patients achieving remission after influenza virus infections (11, 12). Renewed interest in OVs emerged during the 2021 COVID-19 pandemic, when a 61-year-old British man with advanced Hodgkin’s lymphoma experienced tumor regression following COVID-19 pneumonia (13). In 2015, the Food and Drug Administration (FDA) granted approved the first and only OV therapy, T-VEC, a modified herpes simplex virus, for advanced melanoma (14). **Table 1** provides a summary of currently approved OV therapies worldwide.

**Table 1:** Currently approved oncolytic virus products worldwide.

Name	Virus	Country (approval Time)	Indication
Rigvir^*^	ECHO-7	Latvia (2004)	Stage I–II melanoma (15)
H101	AdV-5	China (2005)	Nasopharyngeal carcinoma (16)
T-VEC	HSV-1	USA (2015)	Stage IIIB–IV melanoma (17)
Delytact	HSV-1	Japan (2021)	Glioblastoma (18)

ECHO-7, wild-type echovirus type 7; AdV-5, recombinant adenovirus type 5; HSV-1, recombinant herpes simplex virus type 1; T-VEC, also known as Talimogene laherparepvec; Delytact, Teserpaturev/G47△; * Rigvir has been discontinued in 2019.

There are an estimated 1.5 million undiscovered viruses globally, with around 827,000 are thought believed to be capable of spilling into humans (19). The biological feature of virus determines its ability to infect host cells and replicate under permissive conditions (20). A deeper understanding of viral entry, replication, and their interactions with host immune responses has driven interest in using viruses to treat certain cancers (**Tables 1**, **2**). Although the precise mechanisms of OV therapy are not yet fully understood, it is generally accepted that OVs exert their antitumor effects through direct oncolysis and stimulation of systemic antitumor immune responses (21). 

**Table 2:** Examples some major oncolytic viruses in research and biological features.

Virus	Genotype	Genome length	Entry receptor	Replication site	Associated studies^*^	Ref
Herpesvirus	dsDNA	150kb	HVEM/nectin-1/2	Nucleus/cytoplasm	T-VEC/Delytact/OH2	(22)
Adenovirus	dsDNA	36kb	CAR/CD46/DSG2/Integrins	Nucleus/cytoplasm	H101/ONYX-015	(23–25)
Vaccinia virus	dsDNA	192kb	Receptor-mediated endocytosis	Cytoplasm	GL-ONC1/JX-594	(26–28)
Parvovirus	ssDNA	5kb	SARs	Nucleus/cytoplasm	H-1PV	(29, 30)
Reovirus	dsRNA	123kb	Receptor-mediated endocytosis/JAM-A	Cytoplasm	Reolysin	(31)
Coxsackievirus	ss(+)RNA	7.4kb	CAR/ICAM-1/DAF/KRM1/SCARB2	Cytoplasm	V937	(32–34)
Seneca Valley virus	ss(+)RNA	7.3kb	TEM8	Cytoplasm	SVV-001	(35, 36)
Poliovirus	ss(+)RNA	7.5kb	CD155	Cytoplasm	PVS-RIPO	(35, 37)
Newcastle disease virus	ss (–)RNA	15kb	SARs	Cytoplasm	MTH-68/H/NDV-HUJ	(38)
Measles virus	ss (–)RNA	16kb	CD46/SLAM/nectin-4	Cytoplasm	MV-NIS	(39–41)
Vesicular stomatitis virus	ss (–)RNA	11kb	LDLR	Cytoplasm	rVSV-ZEBOV	(42, 43)

dsDNA, double-stranded DNA; ssDNA, single-stranded DNA; dsRNA, double-stranded RNA; ss(+)RNA, positive-sense single-stranded RNA; ss(-)RNA, negative-sense single-stranded RNA; HVEM, herpesvirus entry mediator; CAR, coxsackie-adenovirus receptor; DSG2, desmoglein-2; SARs, sialic acid residues; JAM-A, junctional adhesion molecule A; ICAM-1, intercellular adhesion molecule 1 or CD54; DAF, decay-accelerating factor or CD55; KRM1, Kringle-containing transmembrane protein 1; SCARB2, scavenger receptor class B member 2; TEM8, tumor endothelial marker 8; SLAM, signaling lymphocyte activity molecule; LDLR, low-density lipoprotein receptor. * In addition to the OVs products approved in **Table 1**, others are still in the preclinical or clinical trial stage.

In this review, we explore the application of viral therapy in lung cancer, focusing on key oncolytic viruses (OVs) and their entry receptors. We then highlight critical signaling pathways and molecules that may synergize with OVs to modulate the tumor immune microenvironment. Finally, we discuss combination strategies, routes of administration, and address biosafety concerns and limitations of virotherapy. In conclusion, virotherapy holds significant promise in advancing lung cancer treatment.

## 2 Oncolytic viruses in lung cancer treatment and entry receptors

### 2.1 Types of OVs in use

To date, many OVs and engineered viral vectors including adenovirus, herpesvirus, vaccinia virus, coxsackievirus, reovirus, poliovirus, Seneca Valley virus, measles virus, and etc., have progressed to early-phase clinical trials (**Table 2**). Currently, at least 6 oncolytic viruses are being evaluated in clinical trials for lung cancer (**Table 3**). The development of OVs for lung cancer therapy is primarily concentrated in the United States, China, and Europe, with clinical investigations limited to phase I-II trials. No successful phase III trials have been reported thus far.

**Table 3:** Current clinical trials of lung cancer-related OVs.

Virus (Submission Time)	Registration Number	Modification	Tumor type	Phase	Location
Adenovirus					
YSCH-01(2021)	NCT05180851	Recombinant L-IFN adenovirus	Lung Cancer	I	China
MEM-288(2021)	NCT05076760	Chimeric IFNβ/CD40-ligand	NSCLC	I	USA
CAdVEC(2018)	NCT03740256	Unknown^*^	Lung Cancer	I	USA
ADV/HSV-tk(2016)	NCT03004183	Replication-defective recombinant adenovirus vector	NSCLC	II	USA
Ad/MG1-MAGEA3 (2016)	NCT02879760	E1/E3 deletion/hMAGE-A3 insertion	NSCLC	I/II	Canada
Colo-Ad1(2014)	NCT02053220	Chimeric Ad11/3 group B	NSCLC	I	Spain
Herpesvirus					
R130(2023)	NCT05886075 NCT05961111 NCT05860374	anti-CD3 scFv/CD86/PD1/HSV2-US11 insertion	Lung Cancer	I	China
T3011(2022)	NCT05598268	Unknown^*^	Lung Cancer	I/II	China
Vaccinia virus					
BT-001(2021)	NCT04725331	Chimeric 4-E03/GM-CSF	NSCLC	I/II	Belgium/France
JX-594(Pexa-Vec) (2008)	NCT00625456	GM-CSF insertion, TK disruption	Lung Cancer	I	Canada/USA
Coxsackievirus					
V937(2014)	NCT02043665	None	NSCLC	I	USA/Australia/UK
Reovirus					
REOLYSIN^®^(2009)	NCT00861627	None	NSCLC	II	USA
Seneca Valley Virus					
SVV-001(2006)	NCT00314925	None	Carcinoid Neuroendocrine	I	USA
Unknown^*^					
RT-01(2022)	NCT05205421	Unknown^*^	Extensive-Stage SCLC	I	China

NSCLC, non-small cell lung cancer; SCLC, small cell lung cancer; *Data not published or not retrievable. Data were collected from National Clinical Trials (https://clinicaltrials.gov/).

### 2.2 Entry receptors in lung cancer as a prerequisite for the oncolytic effects

**Figure 2:**
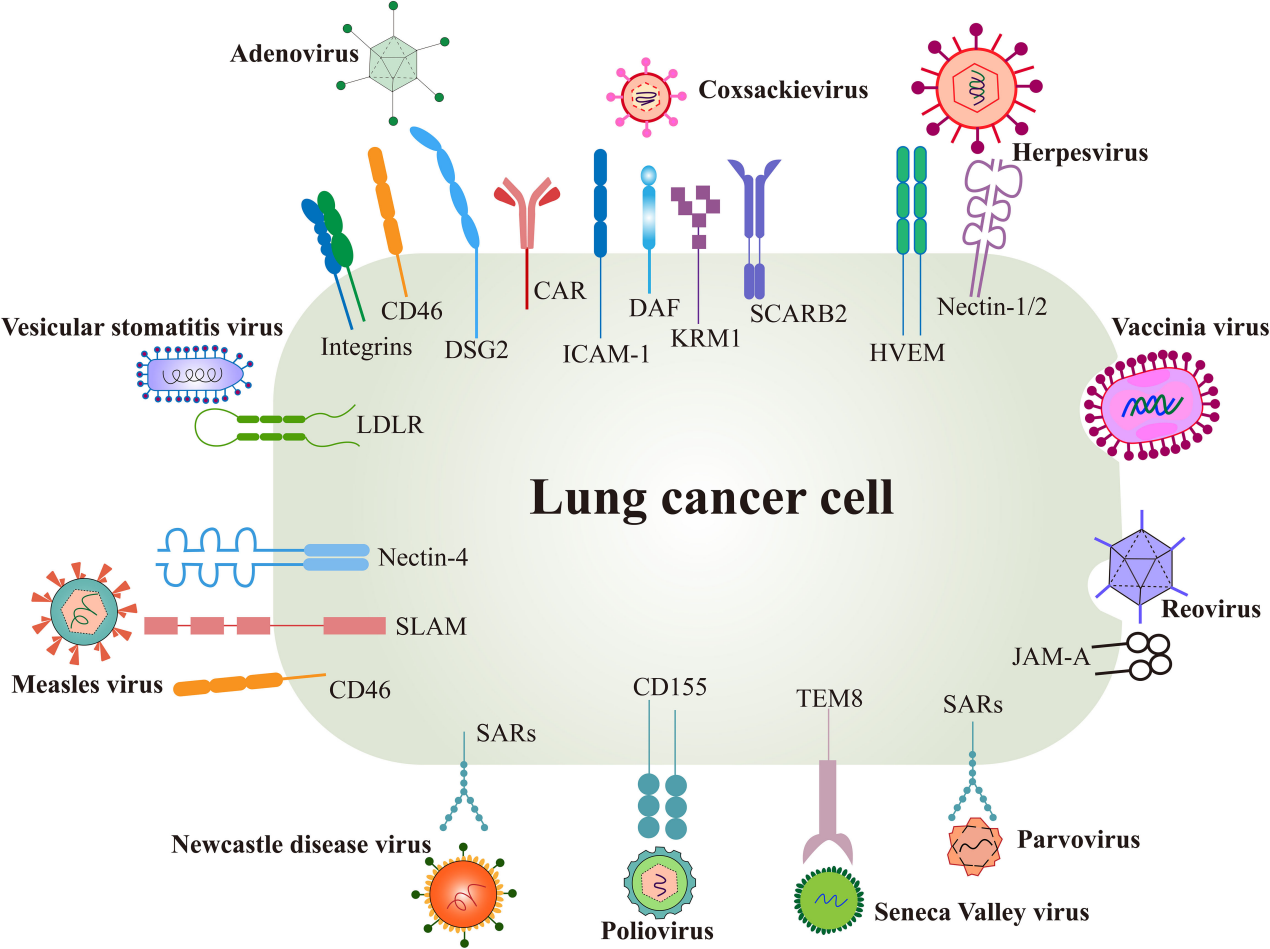
Surface receptors of oncolytic virus entry into cancer cells. OVs utilize several natural receptors to infect host cells, some of which are often overexpressed on lung cancer cells. However, more effective entry targets for OVs remain to be identified. Created using Adobe Illustrator 2024, with data referenced from published literature.

Surface receptors are the first switch that mediate viral entry and determine the viral tropism to tumors. The interaction between viral glycoproteins and host cell receptors facilitates membrane fusion and subsequent viral replication (44). Each virus has evolved specific mechanisms for genome integration, often binding to multiple receptors, while individual receptors may also be targeted by different viruses, enhancing viral infectivity from an evolutionary perspective (45) (**Figure 2**).

Several surface proteins are overexpressed in certain lung cancer cells, such as the coxsackie-adenovirus receptor (CAR), herpesvirus entry mediator (HVEM), and CD46. These receptors not only promote cancer cell invasion and metastasis but also serve as natural targets for OV infection (46, 47). Conversely, low receptor expression can limit the efficacy of oncolysis.

Differences in viral receptor expression provide opportunities for OVs modification. For instance, viral capsid proteins can be modified with peptide ligands or antibody fragments to target specific receptors. Adenoviral capsid fibers have been modified with an Arg-Gly-Asp (RGD) motif to bind integrins overexpressed in tumors, significantly enhancing the targeting efficiency of oncolytic adenovirus type 5 (48). However, limited data exist on the preclinical and clinical characterization of these modifications, highlighting the need for further investigation into receptor expression and associated signaling pathways in lung cancer cells to identify OVs with enhanced tropism.

#### 2.2.1 Adenovirus

Adenovirus (Ad) is non-enveloped viruses, 90-100 nm in size, with a genome contained within an icosahedral capsid (49). There are 57 known Ad serotypes, with Ad2 and Ad5, both from subtype C, the most widely used for OVs (50, 51). Ad is considered a promising oncolytic virus due to its wide range of serotypes and receptors, high titer production, genomic stability, feasibility of genetic modification and well-characterized replication (52).

**Figure 3:**
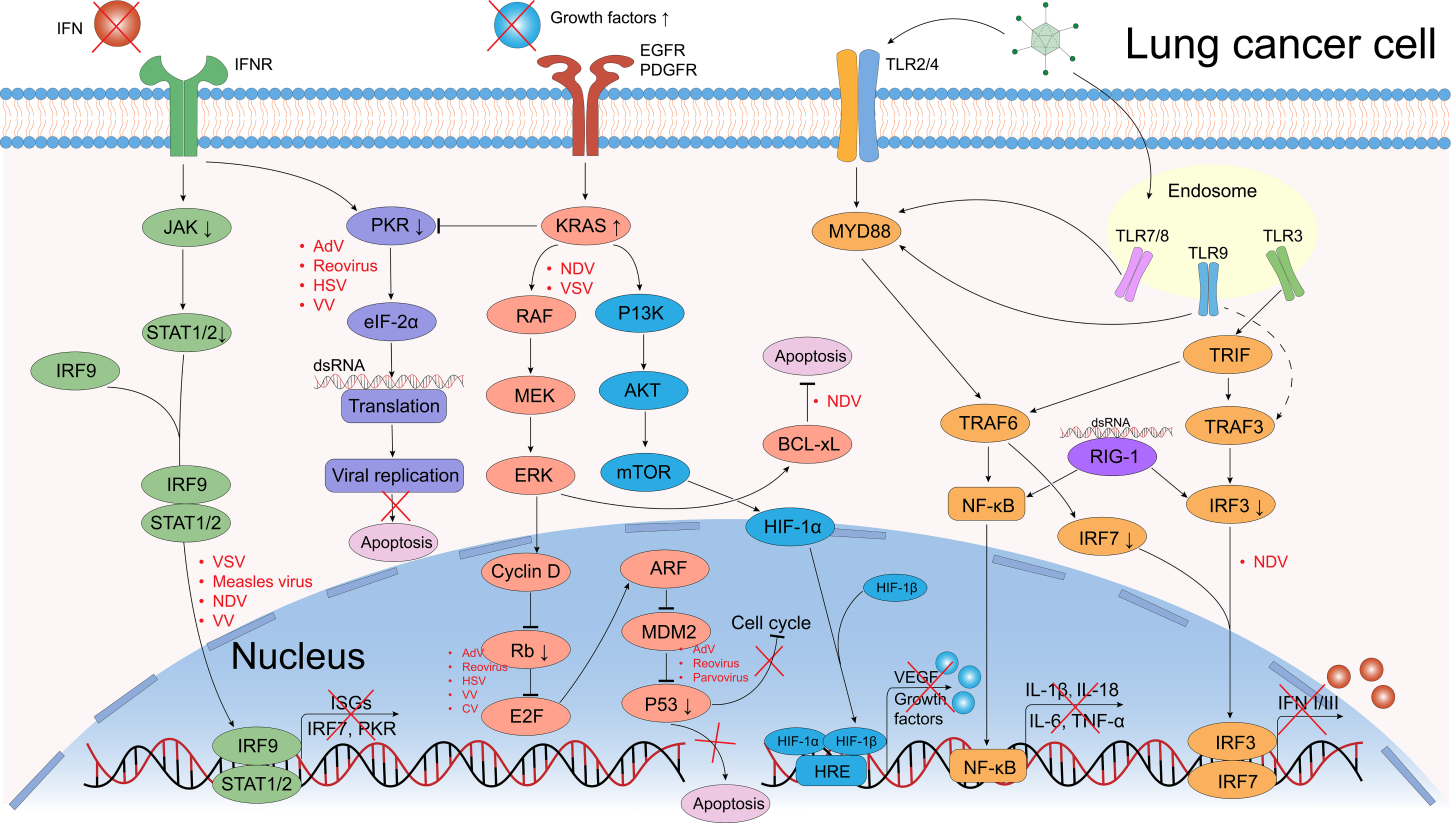
Defective antiviral responses and aberrantly activated signaling pathways OVs can use in lung cancer cells. The clearance of OVs in normal cells depends on the regulation of IFN-related signaling pathways, however, they are frequently dysregulated in cancer cells, which provide great convenience for the OVs’ replication. In normal cells, when viral components are recognized by Toll-like receptors (TLRs) or retinoic acid-inducible gene 1 (RIG-1), they further activate the transcription and translation of downstream NF-κB and interferon regulatory factor (IRF) signals, causing the release of pro-inflammatory factors (IL-1β, IL-18, IL-6, TNF-α) and interferons (IFNs). IFNs bind to IFN receptors (IFNR), activating the JAK/STAT pathway, which induces interferon-stimulated genes (ISGs) expression and further IFNs production. Dysregulation of tumor suppressor genes such as p53 and Rb can promote OV replication (e.g., Ads, reovirus). The PKR pathway regulates transcription and can induce abortive apoptosis in response to viral infection. The EGFR-KRAS pathway is frequently dysregulated in lung cancer, making these cells susceptible to OVs like NDV and VSV. Similarly, the PI3K/AKT/mTOR pathway could upregulate the HIF-1α expression that promote the transduction of vascular endothelial growth factor (VEGF) and growth factors, a certain VV could target the VEGF and generates anti-angiogenic effects. Created using Adobe Illustrator 2024, with data referenced from published literature.

The coxsackie and adenovirus receptor (CAR) is a 46 kDa transmembrane glycoprotein in the junction adhesion molecule (JAM) family and serves as a common mediator for both coxsackieviruses B and most Ads (53) (**Figure 2**). CAR plays a crucial role in epithelial cell adhesion and signal transduction, as well as cancer development (44). Notably, CAR is rarely expressed on normal lung cells but shows variable levels of expression on lung cancer cells (54). H101, with E1b55K and partial E3 deleted, infects cells by the binding of the viral fiber knob with CAR (55). Deletion of E1b55K allows Ad preferentially replicate in p53-deficient cancer cells (56) and E3 genes mainly participate in host anti-virus immune response (**Figure 3**), however, the mechanism by which H101 selectively replicates in cancer cells remains uncharacterized. A preclinical study confirmed that the lung adenocarcinoma cell line XWLC-05 from Xuanwei highly expresses CAR by RT-PCR and immunocytochemistry staining, thereby the oncolytic adenovirus H101 is able to efficiently infect XWLC-05 and lead to oncolysis *in vivo* (57). However, the hypoxic environment of some solid tumors is often associated with CAR downregulation, and the RAS-MEK signaling pathway has also been linked to reduced CAR levels (58). Stecker et al. proposed that CAR expression in tumor cells may vary by stage and correlate with tumor aggressiveness, suggesting the need to assess CAR expression and the tumor microenvironment before selecting a viral therapy (59).

CD46, a transmembrane protein, serve as an inhibitor of complement activation and negatively regulates the complement system, as well as the primary receptor for most species B Ad types (60). While CD46 is widely expressed in normal tissues, it is often overexpressed in lung cancer, potentially due to abnormal signal transducers and activators of transcription 3 (STAT3) activation and p53 mutations (61). Additionally, CD46 also protects cancer cells from complement-mediated cell death (62). Studies have shown that CD46 is upregulated in lung adenocarcinomas (A549, Z793) more than in squamous lung cancers (QG56, NCI-H520), but the latter has a relatively higher levels of CAR (46). Other complement inhibitory proteins, such as decay-accelerating factor (DAF, or CD55), also play a role in protecting tumor cells from immune surveillance (63).

Desmoglein-2 (DSG2), another major receptor for Ad, a transmembrane glycoprotein belonging to the cadherin family (20). DSG2 has been shown to be involved in cell-cell adhesion and tumorigenesis, and is also overexpressed in NSCLC (64). Sun et al. analyzed lung adenocarcinoma (LUAD) patients and corresponding normal tissues to assess DSG2 expression. Combining their results with the data from TCGA and Oncomine, showed that high DSG2 expression positively correlates with tumor size, lymph node metastasis and TNM stage (65). A meta-analysis demonstrated that high DSG2 expression is associated with poor overall survival (OS) in NSCLC patients (66). Another preclinical and clinical study found that DSG2 overexpression promoted LUAD cell proliferation and migration, potentially through the regulation of EGFR and Src phosphorylation, activation of the PAK1 signaling pathway, and alterations in the tumor microenvironment (TME). DSG2 overexpression was also associated with increased resistance to the EGFR tyrosine kinase inhibitor Osimertinib. However, subsequent studies confirmed that DSG2 expression was not statistically associated with tumor size, differentiation, lymph node metastasis or stages (67), which contradicts the fundings of Sun et al.

Additionally, Ad can use integrins as entry receptor, others like SARs, CD80 and CD86 (61), MHC-I (68), etc. The receptors mentioned above are representative examples. Ad uses a variety of receptors for cell entry, lacking inherent specificity for tumor cells.

##### 2.2.1.1 Genetic modification strategies of adenovirus in clinical trials

Ads utilize a range of receptors to enter cells, which gives them high tissue tropism but limits their specificity in targeting lung cancer cells. Ads are the most commonly used virus in tumor treatments due to various modifications for tumor cell targeting, making it a promising candidate for lung cancer oncolytic therapy (51). Similar to adenoviral engineering strategies, the basic principles of oncolytic viral modification strategies include deletion of pathogenic genes, enhancement of viral tropism and integration of immunostimulatory factors (69).

The pathogenicity of Ads is primarily linked to genes in the E region, which are activated early in the replication cycle and regulate viral replication, cell cycle control, and immune evasion (57). Consequently, in the design of oncolytic adenoviruses, the E1 region is frequently modified or deleted to reduce adenoviral pathogenicity and enhance safety, such as above mentioned H101 and YSCH-01. YSCH-01 is a kind of recombinant Ad modified in the E1A region and inserted with multifunctional anti-cancer L-IFN gene (**Table 3**). The L-IFN gene will induce tumor lysis and anti-tumor immunity when Ad replicates in lung cancer cells (70). An investigator-initiated trial about YSCH-01 reported an objective response rate (ORR) of 27.3%, a median progression-free survival (PFS) of 4.97 months, and a median overall survival (OS) of 8.62 months. These results indicate preliminary efficacy of YSCH-01 in advanced solid tumors, including 5 lung cancer patients (71). Viral tropism is determined by the interaction between viral surface proteins and host cell receptors. In type 5 adenovirus variant VCN-01, the substitution of the heparan sulfate proteoglycan (HSG)-binding domain with an RGD motif improves infectivity and selectively targets integrins-expressing tumor cells (48, 72). Granulocyte-Macrophage Colony-Stimulating Factor (GM-CSF) promotes the proliferation, differentiation, and maturation of granulocytes (e.g., neutrophils) and macrophages, enhancing immune resistance to infections and tumors (73). OVs carrying the GM-CSF gene, such as JX-594 (**Table 3**), destroy cancer cells through replication-dependent lysis and activation of antitumor immune responses.

#### 2.2.2 Herpesvirus

Herpesvirus (HSV) is large enveloped dsDNA virus, with 9 known types, including the commonly studied HSV-1 and HSV-2 from the α-Herpesviridae family (74). HSV replicates in cellular nucleus without integrating into the host genome (75), a feature that enhances its safety profile as an OV.

HSV-1 glycoprotein D and B mediate viral entry by binding to specific receptors on the surface of cancer cells (76). gD serves as a ligand protein for most α-Herpesviridae receptors, which binds to 3 primary receptors: herpes virus entry medium (HVEM), nectin-1, and 3-O-sulfated heparan sulfate (3-OS-HS) (77, 78). gB is a trigger protein that is responsible for viral fusion. Reported receptors binding to gB and mediate entry including paired immunoglobulin-like type 2 receptor-α (79), myosin-9 (80), myelin-associated glycoprotein (81). However, the exact mechanisms by which gB receptors mediate viral entry remain incompletely understood and only a few studies have characterized myosin-9 and myelin-associated glycoprotein in NSCLC (82, 83).

HVEM, belonging to the tumor necrosis factor receptor (TNFR), is expressed predominantly on immune cells, and functions as a primary receptor for HSV-1 and HSV-2, excluding other α HSVs (84, 85). Notably, Ren et al. evaluated 527 NSCLC samples and 56 NSCLC cell lines, suggest that HVEM is overexpressed in NSCLC patients (positive rate 18.6% & 48.2%) with N2 lymph node metastasis or advanced stages, but its expression levels is not capable to predicting OS (86, 87). HVEM is a key immune checkpoint, interacting with B and T lymphocyte attenuator (BTLA) and CD160 (BY55) to trigger inhibitory signals (47). It is independent from PD-1/PD-L1 network and may contribute to immune evasion in lung cancer, making it a promising therapeutic target (88).

Nectin-1 is a cell adhesion protein belonging to the immunoglobulin superfamily, which functions as an entry receptor for a big part of α HSVs (89). The nectin family mainly consists of nectins 1-4, but not much research has been done on how HSV infects cells with the help of nectin-1 or its expression in lung cancer cells. In contrast, nectin-4, a receptor for Measles virus, has demonstrated significant predictive and applied value (90). Nectin-2 also facilitates the entry of certain HSV strains and it might be used in lung cancer diagnosis since high nectin-2 expression in LUAD has been found to be associated with recurrence after surgery (91–93).

HSV-1 was the first oncolytic virus to be genetically engineered for therapeutic use. T-VEC, based on HSV-1 JS-1 strain, is modified to the deletion of neurovirulence factor ICP34.5 and ICP47 genes, also armed with GM-CSF gene, which enhancing the recruitment of APCs and immune filtration of TME (94). OH2 is a novel OV derived from HSV-2 HG52 strain, with the same modification strategy as T-VEC (95). HSV-2-based OVs for lung cancer remain in preclinical development, with no evidence distinguishing HSV-1 from HSV-2 in this context (96, 97).

#### 2.2.3 Vaccinia virus

Vaccinia virus (VV) is a large dsDNA virus from the poxviridae family, approximately 192 kb in length, with a characteristic asymmetric brick-like complex structure (69). It can enter the host cells through membrane fusion or via receptor-mediated endocytosis in acidic environments, though the involvement of a specific host cell receptor remains unclear (26, 98). VV is considered a safe oncolytic agent, as demonstrated by its successful use in smallpox vaccines and its cytoplasmic replication, preventing host genome integration (98).

VV’s tumor selectivity depends largely on the thymidine kinase (TK) gene, which supports viral replication. Since TK is overexpressed in cancer cells and minimally expressed in normal somatic cells, oncolytic VVs with TK deletions have been engineered. Additionally, VV-infected tumor cells secrete viral proteins that activate the EGFR-RAS pathway, further promoting TK synthesis and enhancing VV replication (27).

In addition, VV’s large genome can accommodate substantial exogenous DNA without impairing its replication capacity (99). JX-594, the most well-known VV derivative, has been engineered with a GM-CSF gene insertion and TK deletion, showing promise for intravenous administration (73). Moreover, VV holds significant potential for future tumor vaccine development, particularly due to its ability to deliver therapeutic genes and stimulate robust immune responses (100).

#### 2.2.4 Coxsackievirus

Coxsackievirus (CV), a member of the Picornaviridae family, is a positive-sense single-stranded RNA virus with a genome of approximately 7.4 kb, encapsulated by icosahedral capsid proteins (101). CV possesses several features that make it a promising candidate for lung cancer therapy, including multiple receptor targets, ease of genetic modification, cytoplasmic replication, and the ability to specifically target hypermutated molecular pathways in lung cancer (102).

Kirsten rat sarcoma homolog (KRAS) mutations are present in approximately 30% of NSCLC patients, with 20%-40% in LUAD and only 5% in squamous NSCLC (5, 61). CV-B3, a well-studied oncolytic virus, effectively targets KRAS-mutant LUAD cells (A549, H23, H2030) with minimal effects on normal lung epithelial and EGFR-mutant LUAD cells (H1975, PC-9, H3255, H4006) (103, 104). However, CV-B3 can cause severe viral myocarditis and pancreatitis, limiting its therapeutic potential, and further genetic modifications are needed to reduce its toxicity (105).

CVs primarily enter cells via receptors such as CAR, intercellular adhesion molecule 1 (ICAM-1), decay accelerating factor (DAF, or CD55), KRM1 and SCARB2 (scavenger receptor class B member 2, also known as lysosomal integration membrane protein-2, LIMP-2) (32, 33, 106, 107).

As mentioned above, CAR is a common receptor for Ad and CV, and lung cancer cells that express CAR on their surface would be attacked by both of them theoretically. Future studies may need to find the expression of this receptor on more lung cancer cell lines and patient-derived tumor tissue or primary cells to maximize the oncolytic effect of CV that use this receptor to infect lung cancer cells. Deng et al. found that KRAS mutations downregulate CAR expression by activating the ERK1/2 signaling pathway (103).

ICAM-1 (CD54) is an inducible glycoprotein involved in cell adhesion during immune and inflammatory responses (108). CV-A21, is the most researched recently with ICAM-1 (109). Infection with CVB upregulates ICAM-1 expression and increases production of the pro-inflammatory cytokines, including IL-6, IL-8 and TNF-α (110). NSCLC cell lines with high ICAM-1 expression are sensitive to CV-A11-mediated cytotoxicity, while DAF expression levels do not correlate with cytotoxic effects (32, 111). Preclinical and clinical trials showed that the CV-A21-based OVs product V937, which preferentially lyses ICAM-1 upregulated NSCLC cells, is currently in Phase I clinical trials, but the clinical efficacy intravenously administered V937 with pembrolizumab does not appear to be superior to that of monotherapy (34, 112).

DAF/CD55 is a co-receptor that is expressed in both lung cancer and normal lung fibroblast cell lines (113). It has been shown that DAF assists in the entry of ICAM-1 and CAR (114). DAF can act as a lower affinity attachment site, enhancing virus presentation, or as a virus binding site for subsequent higher affinity binding to ICAM-1 and CAR (63, 111). Overexpression or mutations of epidermal growth factor receptor (EGFR) can be detected in 10%-20% NSCLC patients (115). Shao et al. treated H1395 and H322M NSCLC cells with EGF for 24h, and suggest that EGFR activation increases the expression of CD55, but not CD46, upregulated CD55 expression inhibited the complement system and cytokine secretion required for CD8^+^ T cell activation, and CD55 levels were negatively correlated with infiltration of M1 macrophages and CD8^+^ T cells in human lung cancer specimens (n=24), which indirectly promoted tumor growth and could use predicted patient prognosis (116). Therefore, EGFR mutations may enhance CV infection by upregulating CD55, but this also contributes to immune suppression within the tumor microenvironment (TME), which may facilitate tumor progression.

KRM1 is a widespread membrane-anchoring protein located on the cell surface and in the intracellular membrane which is recently identified as an important entry receptor of a major subset of CV-As (117). Most studies on KRM1 focus on its interaction with CV-A10, yet its expression in lung cancer cells and the oncolytic effects of KRM1-targeting CVs remain unexplored, likely due to incomplete understanding of CV-A10’s infection mechanisms and pathogenesis (117, 118).

SCARB2 is a type-III transmembrane protein which mediates the translocation and reorganization of the endosomal/lysosomal compartment membranes (119). However, the expression conditions and the mechanism of how coxsackieviruses use SCARB2 to infect lung cancer cells needs to be further investigated.

#### 2.2.5 Other OVs

Seneca Valley virus (SVV) is a ss (+) RNA virus that selectively infects and kills neuroendocrine SCLC cells (35, 36, 120). Preclinical studies showed that repeated passaging of SVV in SCLC cell cultures enhances its cytolytic activity over time, suggesting increased anti-tumor efficacy (121). Reovirus, an enveloped dsRNA virus (122), infects host cells via receptor-mediated endocytosis, primarily binding to junction adhesion molecule A (JAM-A), which is overexpressed in NSCLC and thus makes it a target for oncolytic virotherapy (31). Additional OVs, including Measles virus (MV) (123), Newcastle disease virus (NDV) (38), Vesicular stomatitis virus (VSV) (124), and Semliki Forest virus (SFV) have been explored for their potential in lung cancer therapy in preclinical settings (125).

In conclusion, OVs can target lung cancer cells by binding to overexpressed surface receptors. Different viruses show varying potential and limitations in lung cancer therapy, and optimizing viral genetic modifications and receptor selection is key to improving efficacy. Ads offer strong tumor-targeting potential due to their multiple serotypes, ease of genetic modification, and ability to use various receptors for cell entry. However, they lack tumor specificity, are prone to be cleared by immune responses, and show off-target effects, including liver accumulation *in vivo*. HSVs avoid host genome integration risk, their large genomes allow for gene insertions and effective immune activation. However, limited expression of key receptors like HVEM and Nectin-1 in lung cancer and potential neurotoxicity remain concerns for clinical application. VV have large genomes capable of carrying exogenous genes, and their cytoplasmic replication avoids integration into the host genome. Their safety is supported by widespread vaccine use, and deletion of the thymidine kinase (TK) gene enhances tumor cell selectivity. However, unidentified specific receptors limit their targeting, and replication depends on the high metabolic state of tumor cells, reducing efficacy in low-proliferation tumors. CVs can target multiple receptors and certain CVs show selective efficacy in KRAS-mutant lung adenocarcinomas. Its small genome allows easy modification, and cytoplasmic replication reduces integration risks. However, it can cause toxic effects like myocarditis and pancreatitis, requiring further modification to minimize side effects. Additionally, receptor expression (e.g., CAR, ICAM-1) varies across lung cancer types, affecting oncolytic efficacy. Other viruses, such as Seneca Valley virus (SVV), have potential for targeting specific lung cancer subtypes (e.g., small-cell lung cancer) and have shown antitumor activity in early studies. However, clinical data are scarce, mechanisms are unclear, and most are in early development, necessitating further validation of their efficacy and safety.

Future research should focus on understanding the molecular mechanisms of viral entry into tumor cells to enhance specificity and reduce toxicity. Additionally, the therapeutic potential of OVs in clinical settings remains underexplored (**Table 3**) and warrants extensive clinical trials to validate efficacy and safety.

#### 2.2.6 Viral receptor expression may change after lung cancer treatment

Notably, OVs are usually used in combination with other therapies, and cancer cells often undergo molecular and phenotypic changes in response to treatment. Changes in surface receptor expression have been observed after radiotherapy or chemotherapy, though results are inconsistent across studies (126–128).

Harrington et al. assessed the combining effects of oncolytic Ad and the external beam radiotherapy, suggested that CAR and integrins were upregulated in colorectal (HCT116) and head-neck (SIHN-5B) cancer cell lines after radiation (129), but Geoerger et al. (130) reported that radiation does not upregulate CAR and integrins expression in glioma cell lines. that radiation CAR and integrins expression in glioma cell lines. Another experimental research also showed radiotherapy fails to increase CD46 receptor expression in glioblastoma for measles virus (131). Wu et al. (126) demonstrated that pre-medication of camptothecin or doxorubicin downregulated the CAR expression in tumor lines including H1299 (lung), HCT 116 (colon) and BxPC-3 (pancreas), this chemotherapy-induced downregulation was observed in patients undergoing chemoradiotherapy prior to colorectal cancer resection. However, Sakhawat et al. showed that CAR expression was enhanced by using cisplatin which improving the infection of Ad in breast cancer cells lines (127). Further research is needed to elucidate receptor expression changes in cell lines and primary tumors following neoadjuvant chemoradiotherapy, and the intracellular signaling pathways mediating these changes.

Therefore, assessing receptor expression before and after combination therapy is crucial to enhancing its clinical benefit, additional studies are required to clarify the impact of combination therapy on OVs receptor expression across different tumor types.

## 3 Key signaling pathways of OVs entry into lung cancer cells

Upregulation of specific surface receptors and dysregulation of key signaling pathways are critical for OVs’ anti-lung cancer activity (102). Upon recognition by entry receptors, OVs selectively replicate in cancer cells and exploit dysregulated signaling pathways. In normal cells, multiple signaling pathways detect and clear viral particles, a mechanism often impaired in cancer cells (**Figure 3**). The first line of anti-viral response defense depends on the interferon (IFN) release producing by endosomal Toll-like receptors (TLRs) associated signaling pathways (132). TLRs, a class of pattern recognition receptors (PRRs), detect conserved pathogen-associated molecular patterns (PAMPs) (133). TLR1-10 were identified in the past decades, every TLR has capability to induce cytokines and activate different innate immune signals (133).

IFNs are classified into three main types: type-I (IFN-I), type-II (IFN-II), and type-III (IFN-III). In normal cells, IFN-I and IFN-III are induced by OVs infection (134). IFN-I is a pro-inflammatory signaling cytokine that consists of two major isoforms, IFN-α and IFN-β, that performs executing cancer immunosurveillance and promotes the remodeling of the TME, while IFN-II is the IFN-γ that is produced by activated NK cells and T cells (132). IFN-III act as an autocrine signal that triggers the production of IFN-α and IFN-λ to enhance the antiviral and antitumor activities of normal cells (135). When multiple TLRs are activated by PAMPs (including viral capsids, DNA, RNA, and viral protein products), and further triggering host cytokine signaling transduction, these factors including myeloid differentiation primary response protein (MYD88), TIR-domain-containing adapter-inducing IFNβ (TRIF), TNF receptor-associated factor (TRAF) family, interferon regulatory factor 3 (IRF3), interferon regulatory factor 7 (IRF7) and retinoic acid-inducible gene 1 (RIG-1), which in turn recruit the downstream kinases (136). IRF3 binding to IRF7 as a dimer, induces the production of IFNs and stimulates the anti-viral responses via autocrine and paracrine methods (137). These IFN-signals further lead to the phosphorylation and activation of Janus family protein kinases (JAK-STAT signaling pathway), which mediate the signals transduction and activate the transcription factor 1 (STAT1) and STAT2 (138). STAT1 and STAT2 form a multimer with IRF9, which transfers to the nucleus and ultimately induces the expression of interferon stimulated genes (ISGs), assisting in the antiviral response (137, 138).

Enhanced IFNs-release induces the activation of the downstream PKR (an intracellular protein kinase that recognizes dsRNA and other viral components) (139, 140) and initiates a cascade of events leading to the phosphorylation of eIF-2α. This phosphorylation inhibits protein synthesis, thereby suppressing further viral replication within cells (141, 142). However, it is possible that the signaling pathways of IFN and PKR are defective in certain cancer cell types, which could result in increased viral replication and impaired viral clearance. Conversely, these pathways may be more active in other cancer cell types, which could impact the therapeutic effect of oncolytic viruses (101, 143).

The mitogen-activated protein kinase (MAPK) cascade (RAS/RAF/MEK/ERK signaling pathway) is frequently dysregulated in cancer and regulates processes like apoptosis, proliferation, and motility (144, 145). The KRAS (Kirsten rat sarcoma viral oncogene) mutant accounts for almost 75% of RAS mutant cancers, which are the most common mutations in NSCLC patients (5, 146). However, despite a long history of preclinical and clinical studies, attempts to develop molecularly targeted drugs against mutations in KRAS in the past decades have ended in failure, therefore, KRAS as a molecular target has been named to be “undruggable”.

KRAS always acts as an intracellular switch, it is activated when bound to guanosine triphosphate (GTP) and inactivated by guanosine diphosphate (GDP)-bound state (147). KRAS-activation further promotes the RAF recruitment and PI3K, which facilitates the process of oncogenesis through downstream effectors (148, 149). Notably, EGFR is proposed to induce the KRAS activation through recruitment and interaction of some growth factor receptor-bound proteins, so upregulated EGFR expression may promote the KRAS activation (149). VV enters cells via receptor-mediated endocytosis, and its replication depends on EGFR-induced RAS signaling, so cancer cells with overexpression of EGFR are more susceptible to VV infection (145) (**Figure 3**).

Furthermore, people have been noted that certain viruses such as coxsackievirus and herpesvirus are capable of selectively targeting cancer cells that exhibit elevated RAS signaling activity. RAS-activated cancer cells fail to activate the PKR pathway, allowing viral infection and oncolysis (103, 150). MEK inhibition disrupts RAF-MEK-ERK signaling, upregulates CAR expression, and enhances adenovirus entry and oncolysis (151). The loss of CAR expression in cancer cells is at least in part mediated by the RAF-MEK-ERK transduction pathway. Restoring CAR expression on the cell surface could enhance Ad-based cancer therapies (152).

PI3K/AKT/mTOR is another RAF/MEK/ERK-independent KRAS downstream signaling pathway, which regulates the expression of hypoxia-inducible factors (HIFs) (153) (**Figure 3**). Jiang et al. (154) showed that NDV triggers autophagy in A549 lung cancer cells resistant to first-line therapeutics (cisplatin and paclitaxel) by targeting the PI3K/Akt/mTOR pathway. Similarly, a recombinant CV-B3 (155) have shown *in vitro* experiments that it induces apoptosis and phosphoinositide 3-kinase/Akt and mitogen-activated protein (MAP)/modulated extracellular signaling (ERK) kinase (MEK) survival signaling pathways, leading to cytotoxicity and modulation of CVB3 replication (104). BCL-xL is a therapeutic target for SCLC and NSCLC (156, 157), an anti-apoptotic protein belongs to the B cell lymphoma (BCL) family of cell survival proteins, and BCL-xL-overexpressed cancer cells permit NDV infection and viral syncytium formation required for viral spread (**Figure 3**) (157).

## 4 OVs induce systemic immune response against lung cancer

Descriptions of the receptors for several major OVs and key signaling pathways provide a general framework for lung cancer therapy. The presence of multiple natural receptors on the surface of lung cancer cells provides targets for viral entry, and defects in the antiviral response and signaling pathways of cancer cells create a microenvironment that supports viral replication, viral elements and cytokines-releasing further lead to an antitumor immune microenvironment (158). Although the specific molecular and cellular mechanisms of OVs are not fully elucidated, they can generally be described in two steps: selective killing of lung cancer cells and induction of an anti-tumor immune response (159, 160).

**Figure 4:**
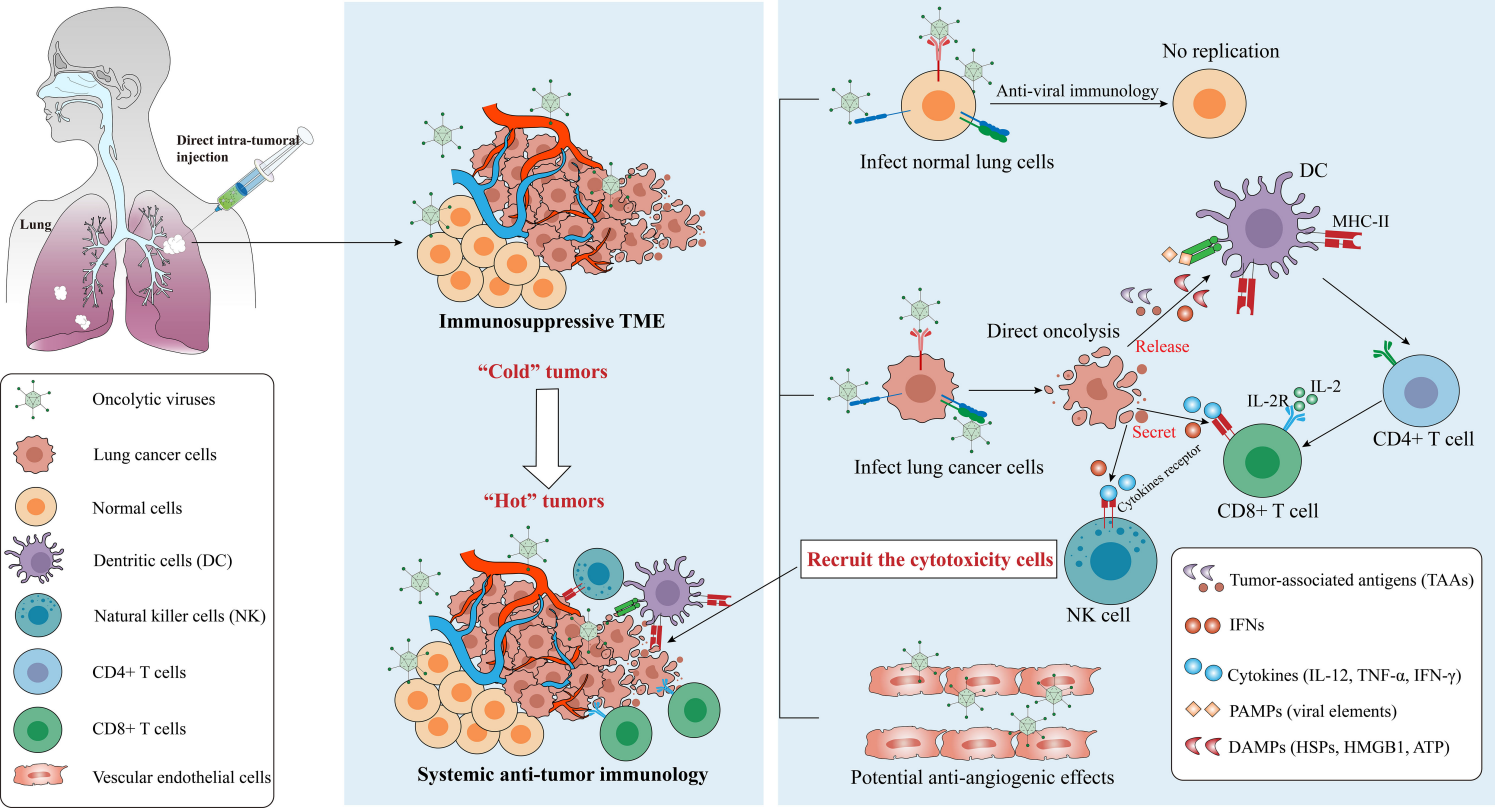
Mechanisms from “Cold” tumor becomes “Hot” tumor. Oncolytic efficacy depends on the selectively killing effect and the activation of anti-tumor immunity, and some OVs have the ability to destroy tumor vascular system. OVs can be cleared by the antiviral responses in normal cells but replicate in cancer cells, which finally leads to the recruitment of cytotoxicity cells such as CD8^+^ T cells via several signaling pathways. Notes: PAMPs, pathogen-associated molecular patterns, include viral capsids, DNA, dsRNA/ssRNA, viral proteins; DAMPs, damage-associated molecular patterns, include HSPs, calreticulin, HMGB1, ATP, uric acid; cytokines include TNF-α, IFNs, IL-12, IL-2, etc. IL-2, interleukin-2; IL-2R, IL-2 receptor; TLR, Toll-like receptor. Created using Adobe Illustrator 2024, with data referenced from published literature.

OVs induce the establishment of acquired immunity and turn “cold” TME into “hot” one (161) (**Figure 4**). OVs infect and replicate in tumor cells which induces an inflammatory response and immunogenic cell death (ICD), for instance, pyroptosis, autophagy and necroptosis are more immunogenic forms of cell death than apoptosis (162, 163). Various forms of ICD are observed following OV infection, which enhances tumor cell oncolysis (164), then a large number of damage-associated molecular patterns (DAMPs) including calreticulin, heat-shock proteins (HSPs), ATP, uric acid, high mobility group box 1 (HMGB1), pathogen-associated molecular patterns (PAMPs) including viral elements, tumor-associated antigens (TAAs) and cytokines (including IL-2, TNF-α, IFN-γ) are released (104, 165, 166). These factors recruit antigen-presenting cells (APCs) to the site of infection, which present antigens to T and B cells, inducing infiltration of cytotoxic T lymphocytes (CTLs), natural killer (NK) cells, CD4^+^ and CD8^+^ T cells (162).

The CD4^+^ T cells function as helpers by secreting cytokines such as IFN-γ, TNF-α, IL-2, and IL-12, which support the activation of CD8+ T cells. These cells are known as well as Th1 cells (167). Once activated, CD8^+^ T cells exert anti-tumor effects locally or by migrating to tumor sites. Meanwhile, NK cells could be activated by IFN-I and DAMPs, and down-regulation of human major histocompatibility complex I (MHC-I) in cancer cells and increased MHC-II expression in APCs also further remodeled the inherent immune response and systemic immune status of TMEs (14, 21). Notably, in addition to the direct killing and TME-reshaping effect to tumor cells, the OVs also shows antiangiogenic effects through killing the tumoral vascular endothelial cells (168, 169), for example, VSV infects and destroys the tumor vascular system *in vivo* but leaves the normal vascular system intact (170). However, circulating OVs face the risk of clearance by neutralizing antibodies. The mechanisms balancing immune-mediated viral clearance and antitumor immune induction require further investigation.

## 5 Combination therapy and route of administration

Given the tumoral heterogeneity, complex genetic mutations, and the immunosuppressive TME, OV-based monotherapy often fails to achieve optimal oncolysis in lung cancer, as demonstrated in most studies (34, 171, 172). Combination strategies involve conventional lung cancer treatments, immune checkpoint inhibitors (ICIs), chimeric antigen receptor (CAR) T-cell therapy, and molecular targeted drugs. For example, H101 has demonstrated oncolytic potential in both *in vivo* and *in vitro* studies, though *in vivo* effects remain relatively mild (57), a meta-analysis shows the overall response rate (ORR) of H101 combined with chemoradiotherapy is significantly higher than those lung cancer patients treated alone, and improving both patient survival rates and quality of life (173). Sei et al. investigated the combination effects of Reovirus type 3 Dearing strain (ReoT3D) and chemotherapeutics in 9 NSCLC cell lines, the results demonstrated ReoT3D combined with paclitaxel can increase the proportion of mitotic blocked and apoptotic cells, and strong oncolytic effects on tumor killing synergized with cisplatin, gemcitabine, or vinblastine (174). A single-arm study for 37 NSCLC patients with metastatic KRAS or EGFR mutations using Reolysin (an OV product based on Reovirus type 3) in combination with paclitaxel and carboplatin, compared favorably (the median progression-free survival (PFS) and overall survival (OS) were 4 months and 13.1 months, respectively) with previous studies for the chemotherapy-alone (171). Cui et al. (54) screened a coxsackievirus B5/Faulkner strain (CV-B5) as an oncolytic virus candidate against NSCLCs (A-549, NCI-H1299, NCI-H460) through *in vivo* and *in vitro* experiments, and CV-B5/F can accelerate cell apoptosis, autophagy and endoplasmic reticulum stress in combination with DNA-dependent protein kinase (DNA-PK) or ataxia telangiectasia mutated protein (ATM) inhibitors. Collectively, pre-clinical and clinical data are still limited and more clinical trials are needed to validate the therapeutic efficacy of OVs and different combination strategies in lung cancer patients.

The method of administration is another major challenge in the clinical application of OVs. The modes of administration of OVs include intra-tumoral (i.t.), intravenous (i.v.), intraarterial (i.a.), and even inhalation (37). Direct i.t. injection is the most studied method, offering advantages such as reduced risk of neutralization by antibodies, more targeted delivery, and localized infection, as for solid tumor in the thoracic, i.t. injections can also be performed by image-guided techniques (41). A study concerning oncolytic VV for malignant pleural effusion caused by NSCLCs, demonstrated that i.t. administration of oncolytic VV was safe and feasible, could produce local immune responses without other significant systemic symptoms (175). However, for metastatic or infiltrative tumors such as neuroendocrine tumors and leukemia, patients may require multiple injections, and OVs may struggle to reach all target tissues. Although systemic methods such as i.v. and i.a. injections are less studied, they offer the ability to treat multifocal or infiltrative tumors with the possibility of repeated administrations (176). i.v. injection may lead to immune clearance of OVs by neutralizing antibodies. However, this issue could potentially be addressed by using extracellular vesicles (EVs) to encapsulate and deliver OVs. Garofalo et al. (177) have validated that human lung cancer cell-derived EVs can be utilized to the delivery of OVs and chemotherapeutics, and its lipid membranes protect OVs from degradation by the immune system. Additionally, inhalation has been explored in viral vaccines, but few studies have investigated OV delivery via inhalation (178, 179), This limited research may be due to concerns over its lower immunogenicity. However, lung cancer usually originates from malignant transformation of bronchial epithelial cells, inhalation administration may be a specific delivery modality for the treatment.

## 6 Biosafety and limitations

Oncolytic viruses, including engineered variants, have demonstrated efficacy as an anti-tumor strategy in numerous preclinical and clinical trials. However, as replicating viruses, OVs raise biological safety concerns related to their potential for replication and infection in non-target tissues. These concerns necessitate careful consideration of storage, handling, and administration protocols (180, 181). Moreover, the toxicity limits of many OVs remain incompletely assessed, and data on potential long-term effects or survival outcomes are still limited. Achieving a balance between antiviral and antitumor immunity is essential for the successful development of OVs. The immune response can restrict viral biodistribution, and OVs are susceptible to detection and inactivation by neutralizing antibodies. Thus, reducing viral toxicity while enhancing antitumor efficacy through genetic engineering and combination with immunotherapy will be crucial future directions. Second, many studies use immunocompromised mice as models, which limits the ability to study virus-immune system interactions in humans. A more rational selection of animal models is needed to better understand the tumor microenvironment (TME) and the interactions among different cellular components.

## 7 Conclusions

Lung cancer is an escalating global public health issue, and its therapeutic strategies are continuously evolving. The emergence of OVs offers a favorable risk-benefit ratio for lung cancer treatment. However, the molecular and cellular mechanisms underlying the oncolytic effects of OVs are not yet fully elucidated.

As previously discussed, several viruses are potential candidates for OVs, with natural receptors on the surface of lung cancer cells serving as therapeutic targets. Future research should focus on identifying more effective targets on the surface of lung cancer cells, elucidating key oncolytic signaling pathways of OVs, and further investigating the TME reshaping process.

In conclusion, while oncolytic virotherapy shows significant promise for lung cancer treatment, additional research is necessary to optimize OVs design and improve clinical efficacy. Combining OVs with other therapeutic modalities could offer a more comprehensive and effective strategy for treating lung cancer.
